# 755 Does Military Service History Make a Difference in Outcomes after Burn Injury?

**DOI:** 10.1093/jbcr/irac012.308

**Published:** 2022-03-23

**Authors:** Callie Abouzeid, Leopoldo C Cancio, Sean A Hickey, Anna DiLisio, Samuel P Mandell, Lauren J Shepler, Barclay T Stewart, Kate E Surette, Steven E Wolf, Lewis E Kazis, Jeffrey C Schneider, Colleen M Ryan

**Affiliations:** Spaulding Rehabilitation Hospital, Boston, Massachusetts; US Army Institute of Surgical Research, JBSA Fort Sam Houston, Texas; Massachusetts General Hospital, Boston, Massachusetts; Shriners Hospitals for Children - Boston, Boston, Massachusetts; UT Southwestern, Parkland Regional Burn Center, Dallas, Texas; Spaulding Rehabilitation Hospital, Boston, Massachusetts; University of Washington, Seattle, Washington; Shriner's Hospitals for Children - Boston, Boston, Massachusetts; University of Texas Medical Branch at Galveston, Galveston, Texas; Boston University School of Public Health, Spaulding Rehabilitation Hospital, Harvard Medical School, Boston, Massachusetts; Spaulding Rehabilitation Hospital, Harvard Medical School, Massachusetts General Hospital, Boston, Massachusetts; Harvard Medical School, Boston, Massachusetts

## Abstract

**Introduction:**

There is little published research on the long-term outcomes of military service members with burn injuries. Military service members are often exposed to extraordinary challenges and adverse experiences that might influence their recovery from a burn injury. Therefore, to better understand the outcomes of service members with burn injuries, this study compares characteristics and health outcomes between burn survivors who have and do not have a history of military service.

**Methods:**

Data from the Burn Injury Model System National Longitudinal Database (2015-2021) were analyzed. Military and civilian groups were identified by the following question administered at hospital discharge: “have you ever served in the military?”. The database does not identify if military service members were active duty at time of injury. Demographic and clinical characteristics as well as outcome measures at one year after injury were compared between groups. Patient reported outcome measures included: Veterans Rand 12-Item Health Survey (VR-12) physical and mental health, Satisfaction with Life Scale (SWL), 4-D Itch, Post Traumatic Stress Disorder (PTSD) Check List, self-reported PTSD diagnosis, and employment status. Multivariate regression analyses examined associations between outcome measures and military service status, controlling for age, gender, burn size, race, and ethnicity.

**Results:**

The study population included 672 burn survivors, 105 with and 567 without military service history, respectively. Participants with military service history were more likely to be male (93% vs. 65%, p < 0.001), older (55 vs. 45 years, p < 0.001) and have Medicare/Medicaid insurance (46.6% vs. 37.2%, p< 0.01) than those without military service history. Race, ethnicity, education, marital status, injury etiology, burn size, length of stay, burns to critical areas, amputation, and number of surgeries were not significantly different between groups. Regression analyses also demonstrated that none of the outcome measures examined (VR-12, SWL, 4-D Itch, PTSD Check List, self-reported PTSD, employment) were significantly different between groups at one-year after injury (p >0.05).

**Conclusions:**

No significant differences in outcomes were found in the BMS Database between burn survivors with and without a history of military service. Harmonization of these data with other military service datasets will help us better understand long-term outcomes of this population.

